# Neuroimaging of Reading Intervention: A Systematic Review and Activation Likelihood Estimate Meta-Analysis

**DOI:** 10.1371/journal.pone.0083668

**Published:** 2014-01-10

**Authors:** Laura A. Barquero, Nicole Davis, Laurie E. Cutting

**Affiliations:** 1 Department of Special Education, Peabody College, Vanderbilt University, Nashville, Tennessee, United States of America; 2 Vanderbilt Kennedy Center, Vanderbilt University, Nashville, Tennessee, United States of America; 3 Department of Radiology and Radiological Sciences, Vanderbilt University School of Medicine, Nashville, Tennessee, United States of America; 4 Vanderbilt University Institute of Imaging Science, Nashville, Tennessee, United States of America; 5 Department of Pediatrics, Vanderbilt University School of Medicine, Nashville, Tennessee, United States of America; University of Plymouth, United Kingdom

## Abstract

A growing number of studies examine instructional training and brain activity. The purpose of this paper is to review the literature regarding neuroimaging of reading intervention, with a particular focus on reading difficulties (RD). To locate relevant studies, searches of peer-reviewed literature were conducted using electronic databases to search for studies from the imaging modalities of fMRI and MEG (including MSI) that explored reading intervention. Of the 96 identified studies, 22 met the inclusion criteria for descriptive analysis. A subset of these (8 fMRI experiments with post-intervention data) was subjected to activation likelihood estimate (ALE) meta-analysis to investigate differences in functional activation following reading intervention. Findings from the literature review suggest differences in functional activation of numerous brain regions associated with reading intervention, including bilateral inferior frontal, superior temporal, middle temporal, middle frontal, superior frontal, and postcentral gyri, as well as bilateral occipital cortex, inferior parietal lobules, thalami, and insulae. Findings from the meta-analysis indicate change in functional activation following reading intervention in the left thalamus, right insula/inferior frontal, left inferior frontal, right posterior cingulate, and left middle occipital gyri. Though these findings should be interpreted with caution due to the small number of studies and the disparate methodologies used, this paper is an effort to synthesize across studies and to guide future exploration of neuroimaging and reading intervention.

## Introduction

Young children who are at-risk for reading difficulties (RD) lag behind their peers in academic achievement and this achievement gap may grow over time [1], [2], resulting in a lifelong condition that negatively impacts academic achievement, employment opportunities, and social interaction. Consequently, remediating reading difficulties is a paramount goal of education, requiring research that disentangles the causes and symptoms of reading difficulties and develops effective treatment. RD can involve difficulties with reading at the word level or language processing level. While some children have specific comprehension difficulties, children with word level difficulties (decoding and/or word recognition) may have comprehension difficulties as well. In the general population, estimates of reading difficulty in children at the word level (dyslexia) range from 6 to 17% [3] and these children typically have a phonological processing deficit (e.g., [4]). Children with word-level difficulties tend to be identified during the early elementary years [5], [6], though some do not experience difficulties until late elementary or middle school years [7]–[10].

### Behavioral Responsiveness to Intervention

Behavioral interventions have been found effective in remediating reading difficulties in some, but not all, individuals with RD [4], [11]–[16]. Interventions that are effective for most children involve explicit instruction and address the reading components of phonological awareness, phonics, fluency, vocabulary, and comprehension [17]–[20]. Determining which children will and will not readily respond to intervention could inform resource allocation such that children who are not likely to respond well could receive more intensive or individually targeted instruction sooner. A review by Al Otaiba and Fuchs [12] determined that the majority of children who exhibit low responsiveness to intervention have a phonological awareness deficit, and other deficiencies may include problems with phonological retrieval or encoding, verbal ability, behavior, and developmental delays. These findings are supported by meta-analyses showing that individual responsiveness to intervention can be influenced by rapid automatic naming (RAN) skills, problem behaviors, phonological awareness, alphabetic principle, memory, and IQ [21], and that pre-intervention differences in real word identification, word attack, and reading comprehension are predictive of gains following intervention [22]. These studies establish the behavioral characteristics of deficient responsiveness quite well, but perhaps these are overt symptoms of an underlying deficit. Biological factors (genes and the brain) influence cognitive factors which in turn influence behavior, all three of which are impacted by an individual's environment [23]. Understanding the behavioral profile of differential intervention response is necessary and useful, but to further characterize RD and potential underlying causes, the neurobiology of reading, RD, and responsiveness to intervention should be explored.

### Neurobiology of RD and Learning

Though extensive literature has characterized the behavioral aspects associated with RD, the underlying cognitive causes of RD are not fully elucidated and are currently under investigation. RD in the general population appears to be part of a normally distributed continuum of reading ability [24], [25]. Growing evidence indicates that the behavioral symptoms of RD are associated with anomalous underlying neurobiology which may be distributed along a continuum as well or perhaps diverges from that of typical readers in different ways according to the type of reading deficit as some structural evidence suggests [26]. This underlying neurobiology is likely influenced by multiple factors—genetics [27], training/instruction [28], [29], nutrition [30], epigenetics [31], [32] and possibly other factors [33]. The interplay of these factors creates variability in RD and adds complexity to diagnosis and treatment. Understanding the learning process at the neurobiological level and how this process may differ for individuals with RD may improve outcomes in the future for those with RD.

#### Neuroplasticity

The capacity to develop neural pathways and adapt to cognitive demands is the essence of neural plasticity. Changes in electrical impulses, chemical signaling, and histology underlie the behavioral aspects of “learning.” Describing the candidate mechanisms of neuroplasticity in detail is beyond the scope of this paper but to illustrate the complexity of processes involved in neuroplasticity we describe some of what is known and refer the reader to several excellent review papers. At the synaptic level, long-term potentiation (LTP) of the postsynaptic neuron in response to repeated presynaptic stimuli is widely believed to be a primary mechanism of long-term memory and learning [34]. LTP can lead to synaptic modifications that result in enhanced signal transmission [35]. Though short-term synaptic modification does not require protein synthesis, long-term synaptic changes seem to require protein synthesis and these long-term changes are considered to be the cellular correlates of learning and memory [36]. Beyond enhancement of synaptic connections through LTP, gray matter structural mechanisms of neuroplasticity include neurogenesis, axon sprouting, dendritic branching (and synaptogenesis), gliogenesis and glial modifications, and angiogenesis [37]. As numerous gene products are believed to be involved in learning and memory and protein synthesis appears to be required for long-term memory storage, regulation of gene expression is likely to be important in learning processes. Epigenetic mechanisms include histone modification [31] and post-transcriptional regulation of gene expression by microRNAs [38]. Clearly, learning and memory are complex events at the cellular and molecular levels. Yet, what does this mean for children with learning disabilities? Perhaps these processes differ for individuals with RD compared to typically achieving peers. An inefficiency in one mechanism of the intricate processes involved in storing and retrieving information could adversely affect learning. Such neurobiological differences could explain why some children absorb new knowledge effortlessly while others not only struggle to grasp new concepts, but may also have great difficulty in retaining and consolidating information.

If a characterizable neurobiological anomaly underlies poor response to instruction, identifying these individuals and developing an appropriate intervention could possibly compensate for the deficiency. To illustrate, a critical molecular process related to long-term memory is the effect that CREB (cAMP response element binding protein) has on genetic transcription. CREB is stimulus-inducible in neurons and involved in long-term memory by influencing protein synthesis [39]. In a mouse study, long term memory was adversely affected by CREB gene disruption, yet interestingly the learning deficit was overcome by increasing the time between the training events [40]. That is, modifying the behavioral training compensated for a molecular deficiency. Perhaps identifying underlying dysfunction in functional activity can lead to a specific intervention. A challenge lies in understanding these differences and addressing them perhaps in a very prescriptive manner. Whatever the deficiencies may be, characterizing those deficiencies through investigative techniques is a step in the process toward targeted remediation.

#### Functional neuroimaging

Neuroimaging is a tool that can be used to explore brain activity and can make a valuable contribution to understanding neuroplasticity. Brain activity, as determined by imaging modalities such as fMRI and MEG, not only indirectly reflects the underlying tissue structure and physiology (as neurons must be present and functioning to exhibit activity), but also represents which areas of the brain are actively engaged when presented with stimuli of interest. Numerous studies have used imaging techniques to explore brain activity associated with specific cognitive tasks. A growing number of studies are exploring various aspects of reading to determine how the brain accomplishes the complex task of reading. Further investigations are concerned with how the brain differs in functional activity between skilled readers and readers who struggle.

#### Neuroimaging in typically achieving readers

Studies with unimpaired adult readers have revealed a left hemisphere reading network comprised of three areas: a ventral posterior region, a dorsal posterior region, and an anterior region [41]–[44]. The posterior ventral region is located in an inferior occipito-temporal area. This area appears to be involved in visual processing and recognition of words [41], [45]–[48] as even letters and pseudoletters elicit a response in this region [49]. The posterior dorsal region is comprised of the posterior superior temporal gyrus (pSTG), supramarginal gyrus (SMG), and angular gyrus (AG) and appears to be involved in phonological processing, transforming orthographic representations to phonological representations, and semantic processing [41], [47], [50], [51]. The anterior region is located in and around the inferior frontal gyrus (IFG). The IFG appears to be involved in phonological processing (e.g., [49]) and may be involved in articulatory recoding such that phonological input is converted to speech-gesture articulation output [41], [42] and semantic processing [47]. The studies establishing these regions have largely focused on single word reading. However, studies exploring reading comprehension have found left SMG and AG [52] and bilateral middle temporal gyri (MTG) and STG [53] to be activated by typical readers during comprehension tasks. While imaging studies of reading in children indicate a large amount of overlap with adults in activation, there are some differences. Some evidence indicates that over the course of development, activation in some more dispersed areas attenuates with age while increases with age are more focal, and these changes are largely independent of performance [54], [55]. For example, adults show less activation of left SMG and AG, areas involved in phonological processing [56]. This suggests that children are more actively engaged in using phonology to analyze words as they read, whereas adults have developed such automaticity in word reading that increased use of these phonological processing areas is no longer required. In contrast, adults showed increased activity in frontal and parietal regions thought to be involved in attention and top-down cognitive control [54], [55]. It is important to consider developmental changes in functional activity when reflecting on whether reading difficulties are more related to a delay in development or to dysfunctional reading networks that encourage development of compensatory mechanisms. Another recent finding in functional developmental changes is that sensitivity to visually presented words (i.e. activation response to detecting a word among progressively decreasing visual noise) increases over the school-age years in the L posterior occipito-temporal sulcus [57]. This may imply a more refined usage of an area used in visual word reading.

#### Neuroimaging of reading disabilities

Over the past two decades, numerous studies have reported differences in brain function during reading tasks for people with reading problems relative to controls with typical reading achievement and several narrative reviews have highlighted the commonalities among studies [41], [43], [58]–[61]. The functional differences between RD and typical readers are generally characterized by reduced activity in L hemisphere regions for RD. Reviews of the literature have indicated that RD involves a dysfunction of the aforementioned three-region reading network: general underactivation of L temporo-parietal region (including STG) and ventral occipito-temporal (including lateral extrastriate, fusiform, and inferior temporal gyrus) and overactivation of the L IFG. This overactivation of the L IFG has been presumed to be due to compensatory articulatory effort and evidence that contradicts this portion of the accepted model has recently emerged [62]. Though less consistent than reduced left hemisphere activation, some studies have also reported increased right hemisphere activation that may signify compensatory activity for people with RD during reading tasks [51], [63]–[66] and this compensatory activity may develop as early as second grade [67]. Adding further complexity, there is evidence that, at times, typical readers exhibit less brain activation in reading areas than do readers with RD. Rimrodt et al [64] found that adolescents with RD activated more than typical readers in the left middle and superior temporal gyri when reading incongruent sentences, perhaps suggesting more effortful processing of nonmeaningful sentences. Pugh et al [68] found through manipulating stimuli that for non-impaired adolescent readers, factors that make the word easier to process were associated with relatively reduced activation. However, for readers with RD facilitative factors were associated with increased activation in the same areas, “suggesting that the LH reading circuitry in adolescent RD is poorly trained but not wholly disrupted” [68]. Less activation may at times be reflective of knowledge consolidation. If cognitive processing is less effortful, then the resulting efficiency may mean less functional activity. Though evidence is limited, some studies have shown that typically achieving novice performers can exhibit increased activation, yet following training or practice, decreased activation is observed [28], [69], [70].

While the value of the narrative reviews cannot be discounted, meta-analysis provides a statistical approach to synthesizing across studies. In a meta-analysis of adults with reading disabilities compared to controls, reduced activation was reported for L hemisphere ventral occipitotemporal cortex, inferior parietal cortex, STG, IFG, and thalamus [71]. Another recent meta-analysis, which included both children and adults with reading disabilities, identified underactivation in the L hemisphere inferior parietal, superior temporal, middle and inferior temporal, and fusiform regions and also reported underactivation in the L IFG that coincided with overactivation in the primary motor cortex and anterior insula [72]. In a meta-analysis that compared adults with RD and children with RD, showed similar results except that temporoparietal underactivation was seen only for adults with RD, not for children with RD [73].These meta-analyses are consistent with the literature reviews in identifying a dysfunctionally underactivating left hemisphere network in RD. However, the meta-analyses indicate the presence of underactivation of the L IFG in RD rather than the overactivation assumed in the narrative reviews [62]. The meta-analysis would appear to constitute a consensus across studies, yet appropriate caution should be used when interpreting meta-analyses. Due to the necessary requirement of coordinates of activation to perform functional meta-analysis, not all relevant studies may be included in the analysis. Hence, both the meta-analytic approach and the narrative literature review provide insight into understanding differences between typical readers and those with RD.

### Neuroimaging of Reading Intervention Literature Review and Meta-Analysis

Arising from the growing evidence of RD brain activity differences are questions surrounding how reading instruction can impact individuals with RD at the neurobiological level. In addition, questions arise regarding the relation of neurobiological differences and responsiveness to intervention. To answer these questions, we conducted a systematic review of the functional imaging literature associated with reading intervention. We sought studies that explored functional activity differences before, during, and after intervention including studies that examined responsiveness to intervention and associated functional imaging. We sought to include recent studies published since earlier summaries and reviews [74]–[77]. In this paper we limit detailed examination to fMRI and MEG, including magnetic source imaging (MSI) which uses MEG in conjunction with MRI. However, it should be noted that in addition to fMRI and MEG, reading intervention has been explored with other methods and modalities. Structural imaging studies have included diffusion tensor imaging (DTI) [78]–[80] and voxel based morphometry (VBM) [81]. Functional activity studies have used event related potential (ERP) [82], [83], magnetic resonance spectroscopy (MRS) [84], [85] and transcranial magnetic stimulation (TMS) treatment [86], [87].

In an effort to quantify changes associated with treatment, we conducted ALE meta-analysis on a subset of experiments contained in the literature review. Meta-analyses have the potential to identify results that are consistent across studies in a more objective and quantitative manner than can descriptive reviews. The meta-analytic process may reveal commonalities across studies that did not command attention in the original studies because they seemed theoretically uninteresting [62]. Since its development [88], ALE has become available as GingerALE on BrainMap.org [89] which has greatly facilitated the use of the meta-analytic approach for functional imaging experiments. Modifications have been made [90]–[92] such that GingerALE version 2.1.1 supports random effects meta-analysis (rather than fixed effects). Also, values generated by ALE are weighted by sample size of each contributing study. According to the User Manual for GingerALE 2.1, the GingerALE process can be summarized in three steps [93]. First, an ALE value is computed for each voxel in the brain and the null distribution of each voxel's ALE statistic is determined. The full width at half maximum (FWHM) value is automatically included as it has been empirically determined [91]. Second, a false discovery rate (FDR) algorithm uses the *p* values from the previous calculation to compute the ALE map threshold. Third, a cluster analysis of the thresholded map is performed based on the selected minimum cluster size. Meta-analyses of neuroimaging of reading disabilities have been published in recent years [71]–[73]. As ALE meta-analysis is necessarily restricted to including only experiments that provide coordinates in normalized space (Talairach or MNI), currently few studies are available for inclusion. This analysis explores these limited studies as a step toward synthesizing across studies in an objective and quantitative manner.

This paper is a review of the literature associated with neuroimaging of reading intervention in children and adults with RD. First, we review the literature regarding brain-based studies of reading intervention. This descriptive review examines studies conducting imaging before and/or after intervention. Next, we present a coordinate-based ALE meta-analysis using a subset of the studies in the descriptive review. This subset consists of the fMRI studies that provided post-intervention scanning data in sufficient detail required for ALE meta-analysis.

## Methods

### Retrieval of Studies

#### Inclusion criteria

To obtain studies that examined brain activity associated with reading intervention, six inclusion criteria were stipulated. First, only peer-reviewed, primary research studies were included. Second, only studies with at least some participants designated as having reading difficulties, reading disabilities, dyslexia, or at-risk status for reading difficulties were included. For studies that used imaging to predict future reading scores, the designation of reading difficulties could be determined at posttest. Case studies were excluded. Third, the reading difficulty must have been idiopathic in nature and not the result of head trauma, stroke, or illness. Fourth, the studies were required to describe reading-related instruction that occurred during the experiment. Though the intended focus was upon intervention, studies in which participants received regular (business-as-usual) instruction rather than implementing an intervention were not excluded. Fifth, the studies were required to include neuroimaging in the modalities of either fMRI or MEG and the experimental design must associate the imaging with the reading instruction. Sixth, the functional imaging task must have been a reading task or a task of reading-related skill (e.g., letter sound matched to visual letter).

#### Searches

**Figure 1:**
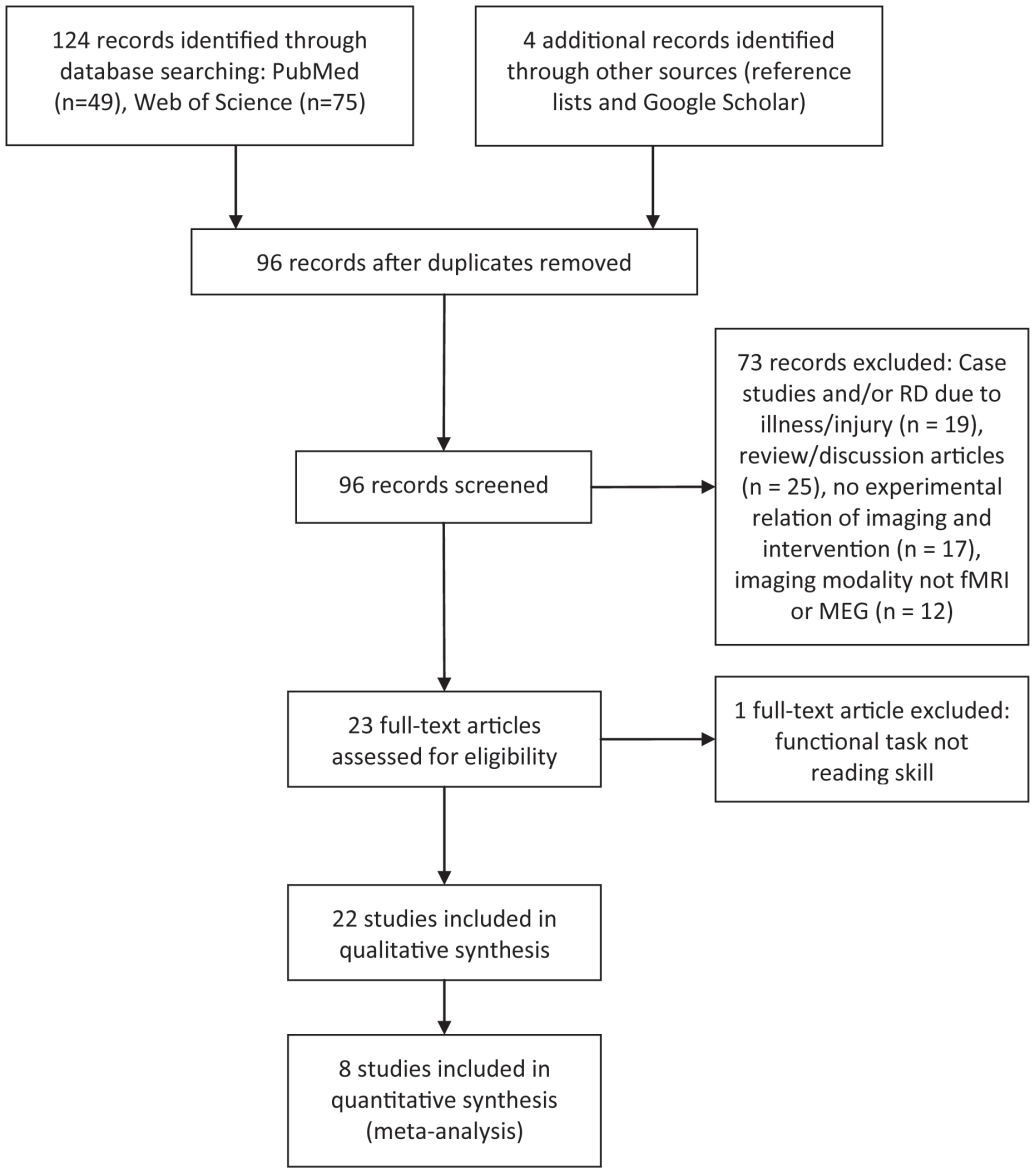
Systematic review flow diagram. (PRISMA template; Moher, Liberati, Tetzlaff, Altman, & The PRISMA Group, 2009).

Two search strategies were employed to identify relevant studies and these searches were current as of January 2013. First, searches were conducted using two electronic databases, *PubMed* and *Web of Science.* The *Web of Science* search input was (TS = ((reading disability OR dyslexia OR reading difficulty) AND (neuroimaging OR fMRI OR brain activation) AND (reading intervention OR reading instruction OR reading treatment))) AND Document Types = (Article). The *PubMed* search input was (((“reading”[MeSH Terms] OR “reading”[All Fields]) AND disability[All Fields]) OR (“reading”[MeSH Terms] OR “reading”[All Fields]) AND difficulty[All Fields]) OR (“dyslexia”[MeSH Terms] OR “dyslexia”[All Fields]) AND ((“neuroimaging”[MeSH Terms] OR “neuroimaging”[All Fields]) OR (“magnetic resonance imaging”[MeSH Terms] OR (“magnetic”[All Fields] AND “resonance”[All Fields] AND “imaging”[All Fields]) OR “magnetic resonance imaging”[All Fields] OR “fmri”[All Fields]) OR ((“brain”[MeSH Terms] OR “brain”[All Fields]) AND activation[All Fields])) AND (((“reading”[MeSH Terms] OR “reading”[All Fields]) AND (“Intervention”[Journal] OR “Interv Sch Clin”[Journal] OR “intervention”[All Fields])) OR ((“reading”[MeSH Terms] OR “reading”[All Fields]) AND (“teaching”[MeSH Terms] OR “teaching”[All Fields] OR “instruction”[All Fields]))). The *Web of Science* search yielded 75 articles. The *PubMed* search yielded 49 articles. The resulting 124 articles were examined and those that did not meet criteria were systematically eliminated (Fig 1) [94]. Next, using references from studies that met inclusion criteria, additional studies were considered. Additionally, Google Scholar searches were performed to locate papers that have cited some of the studies that met inclusion criteria.

### Data collection

Data were gathered from articles, supplemental material, and from other works referenced in the paper as needed (e.g.,detailed descriptions of participants or interventions published separately from imaging analysis). Information describing participant groups, neuroimaging techniques, and whether or not the study only charted reading growth or implemented a reading intervention (in which case reading intervention approach was included) is presented in Results. Of the twenty-two studies included in the qualitative review, 21 included an intervention and 1 only charted reading growth (with no intentional intervention, but some participants received various interventions outside the study). Of the eight studies included in the ALE meta-analysis, all included an intervention.

### ALE meta-analysis

fMRI studies that included either Talairach or MNI coordinates in their results were included in the meta-analysis. Data presented in MNI coordinates were converted to Talairach coordinates using Lancaster conversion provided on brainmap.org [95], [96]. Data presented in Talairach coordinates were included as presented in the original paper regardless of conversion method. Activation likelihood meta-analysis was conducted using GingerALE v.2.1.1 [89], [91], [92], [97]. Only one experiment or contrast per study was included in the meta-analysis such that each participant group is not represented multiple times in analysis. When available, we selected experiments that contrasted activation increases for postscan over prescan in the RD group [63], [98]–[101]. When RD Post > Pre contrasts were not available we used RD Followup > Pre [102]. When only postscan data were available, we used group contrasts RD > Control [69] and Responders > Nonreponders and Controls [103]. Eight experiments were included, with a total of 173 participants and 90 activation foci. Of the 90 foci, 1 fell outside the mask, 1.1% of the total. It is typical for a small percentage of foci to fall outside the mask [93]. A false discovery rate (FDR) of 0.05 and the recommended minimum cluster volume of 264 mm^3^ (generated by GingerALE2.1.1) were used and images were viewed using Mango version 2.6 [104]

It should be noted that we included foci of activation increases that the Eden et al [63] study stated fell short of their significance thresholds of *p*<0.001 and *k*>80. For the Richards et al study[99], we included only the orthographic experimental group as these are the coordinates that were reported and the group that showed significant change in associated with treatment.

## Results

The literature search resulted in 22 studies that met criteria for inclusion in the descriptive literature review (Fig 1). The studies are presented in chronological order with their participant groups, interventions used, and intervention dosages in Table 1. The imaging modalities, functional tasks, and imaging findings are listed in Table 2. Descriptively synthesizing across studies, key findings by anatomical region are presented below. Following this qualitative synopsis, we summarize the findings of the ALE meta-analysis of the eight studies with post-intervention fMRI data.

**Table 1:** Participant groups and interventions.

Study	RD N	CT N	Age	Intervention	Dosage
Simos et al., 2002	8; 6 received Phono-Graphix, 2 received Lindamood Phonemic Sequencing	8	7–17 yrs	Phono-Graphix (Read America, Orlando FL) Lindamood Phonemic Sequencing (Lindamood-Bell, San Luis Obispo, CA)	80 hrs: 1-2 hr/day over 8 wk
Aylward et al., 2003	10	11	139.1 (9.8) months, 137.5 (7.9) months	Instruction in linguistic awareness, alphabetic principle, fluency, and reading comprehension	28 hrs: 2hr/day over 14 session days (3 wk)
Temple et al., 2003	20	12	8–12 yrs	Fast ForWord Language (Scientific Learning Corporation, Oakland, CA)	100 min/day, 5 days/wk, average 27.9 days
Eden et al., 2004	19 total; 9 received intervention	19	adults, RD 44.0 (9.4), CT 41.1 (9.7)	Multisensory instruction including sound awareness, letter-sound association, articulatory feedback administered by Lindamood-Bell Learning Corporation staff	3 hr/day, 8 wks, avg 112.5 hr total
Shaywitz et al., 2004	49 total; 37 received experimental intervention, 12 received community intervention	28	6.1 – 9.4 yrs; RD experimental 7.9 (0.5), RD community 8.1 (0.6), CT 8.0 (0.5)	Experimental intervention [127] included sound-symbol associations, blending, timed reading for fluency, oral reading, dictation	50 min/day for 8 months
Simos, et al., 2005	16; 13 responders, 3 non-responders	17	5.6–7.2 yrs at baseline (Low risk group 5.6–6.5, High risk group 6.0–7.2) 6.4–8.1 yrs at posttest (Low risk 6.4 – 7.5, High risk group 7.0 – 8.1)	Proactive Reading and Responsive Reading [128]	40 min/day, 5 day/wk for 8 months
Richards et al., 2006	18; 8 orthographic treatment, 10 morphological treatment	21	RD 130.8 months, CT 132.6 months	Instruction in alphabetic principle, composition, and either orthographic spelling treatment or morphological spelling treatment	28 hr total: 2 hr/day for 14 sessions over 3 wk
Hoeft et al., 2007	64 struggling readers (identified by teachers, many had scores in average range)	-	10.0 (1.09) yrs	Power4Kids Reading Initiative. Many participants received 1 of 4 interventions, but there was no significant effect of intervention on decoding scores.	about 6 months during school year
Richards et al., 2007	20; 11 phonological treatment, 9 nonphonological treatment	10 nonphonological treatment	RD phonological 137.7 (10.00) months, RD nonphonological 134.60 (11.10) months, CT 128.60 (8.00) months	Phonological treatment included explicit written language instruction using phonological working memory, phoneme-grapheme correspondences in spelling, and science report writing [129]. Nonphonological treatment included nonverbal virtual reality supported science problem solving [130]	24 hrs total—8 sessions over 2 wks with 3 hr/session
Simos, Fletcher, Sarkari, Billingsley-Marshall, et al., 2007	15	-	7–9 years	Phono-Graphix [131] and Read Naturally [132]	16 weeks total: 2 hr/day for 8 wks Phono-Graphix, 1 hr/day for 8 wks Read Naturally
Simos, Fletcher, Sarkari, Billingsley, et al., 2007	15; 8 responders, 7 nonresponders (same as Simos, et al., 2007 above)	10	7–9 years	Phono-Graphix [131] and Read Naturally [132]	16 weeks total: 2 hr/day for 8 wks Phono-Graphix, 1 hr/day for 8 wks Read Naturally
Meyler et al., 2008	23 (possible overlap with Hoeft, et al., 2007)	12	5th grade	Power4Kids project used four programs: Corrective Reading, Wilson Reading, Spell Read Phonological Auditory Training (PAT), Failure Free Reading	100 hrs total over 6 months
Odegard et al., 2008	12 total: 6 responders, 6 nonresponders	6	10 – 14 yrs	Take flight: A comprehensive intervention for students with dyslexia [133]	90 min/day, 4 days/wk for 2 school years
Richards & Berninger, 2008	18 (same as Richards et al., 2006)	21	RD 130.8 months, CT 132.6 months	Instruction in alphabetic principle, composition, and either orthographic spelling treatment or morphological spelling treatment	28 hrs total—14 sessions over 3 wks with 2hr/session;
Davis et al., 2011	10 total: 5 responders, 5 nonresponders	4	7.5 (0.43) yrs	Intervention consisted of sight word reading, letter sound practice, decoding practice, and reading for fluency.	45 min, 3 days/wk, 17 weeks
Farris et al., 2011	10 total: 5 responders, 5 nonresponders (same as Odegard et al., 2008)	5	10 – 14 yrs	Take flight: A comprehensive intervention for students with dyslexia [133]	90 min/day, 4 days/wk for 2 years
Hoeft et al., 2011	25	20	RD 14.0 (1.96) CT 11.0 (2.57)	*This study did not provide an intervention.* 11 participants received some form of intervention, but no differences were observed for intervention.	-
Rezaie et al., 2011a	20 total: 10 Adequate Responders (AR), 10 Inadequate Responders (IR)	20	Adequate Responders 158±7 months, Inadequate Responders 153±11 months, CT 151±11 months	Instruction included word study, fluency, vocabulary, comprehension [134]	45–50 min/day over 1 schoolyear
Rezaie et al., 2011b	27 total: 16 AR, 11 IR (possible overlap with Rezaie, et al., 2011a)	23	Adequate Responders 159±9 months, Inadequate Responders 156±16 months, CT 153±12 months	Instruction included word study, fluency, vocabulary, comprehension [134]	45–50 min/day over 1 schoolyear
Yamada et al., 2011	7 (at-risk)	7 (on-track)	At-risk 5.6 (0.2) yrs, On-track 5.7 (0.3) yrs	Early Reading Intervention [135]	30 min/day, 3 months
Gebauer Fink, Kargl et al., 2012	20 total (poor reading and spelling): 10 Treatment (TG), 10 Waiting Group (WG)	10	10–15 yrs, (*M* = 11.80; *SD* = 1.58)	Morpheus: a computer-aided morpheme-based spelling training in German [136]	Daily handwritten and computer homework, 1/wk instructor-guided courses for 2 hr, over 5 wks.
Bach et al., in press	6 poor readers (group classification made at follow-up)	11	Poor Readers 6.33±0.19 yr, Normal Readers 6.35±0.29 yr	Graphogame: a computerized training game teaching grapheme-phoneme correspondences in German [137]–[139]	321.5±124.3 min over 8 wk

**Table 2:** Imaging and Findings.

Study	Imaging	Imaging task	Principle Findings
Simos et al., 2002	MSI, Pre/Post	Pseudoword rhyme-matching	Pre-intervention underactivation of left posterior STG in RD group increased to level of controls at post-intervention. Control group did not change over time. Additionally, RD showed pre-intervention overactivation of R STG.
Aylward et al., 2003	fMRI, Pre/Post	Letter-Phoneme Matching (with Letters Only Matching control task) Comes From Morpheme Mapping (with Synonym Judgment control task)	Pre-treatment RD underactivated in L MFG, IFG, MTG, ITG, R SFG, and bilateral superior parietal regions during phoneme mapping and in L MFG, R superior parietal and fusiform/occipital area during morpheme mapping. No differences between groups at post-scan due to increased activity for RD group and decreased activity for controls.
Temple et al., 2003	fMRI, Pre/Post	Rhyme Letters (phonological), Match Letters (nonphonological), Match Lines (nonletter)	Following treatment, RD had increased activity in L IFG, anterior cingulate, ITG, MTG/angular, hippocampal, and lingual gyri, R anterior cingulate, MFG, insula/IFG, SFG, MTG, posterior cingulate/precuneus, parieto-occipital sulcus, and bilateral anterior thalamus. These increases were not present in CT.
Eden et al., 2004	fMRI, Pre/Post	Sound Deletion (aurally presented words), Word Repetition (aurally presented)	Post intervention, Group × Session interaction revealed increases in L IPL (BA 4 0), intraparietal sulcus (BA 40/7), fusiform/parahippocampal gyrus (BA 37), hippocampal gyrus, thalamus, and MFG (BA 46), R posterior STS/G (BA 22/39), SPL (BA 7), IPL (BA 40), IFG (45/46), inferior postcentral gyrus (BA 43), medial frontal cortex (BA 10/11/47), and inferior MFG (BA 11).
Shaywitz et al., 2004	fMRI, Pre/Post/1yr follow-up	Matching Letter Name (audio) to Letter (visual), Audio tone/Visual symbol control task	Immediately following treatment, RD experimental intervention group showed increased activation compared to RD community intervention in L IFG and MTG and decreased activation in the R caudate nucleus. One year after treatment ended, the RD experimental intervention group had increased activation in bilateral IFG, LSTS, posterior MTG/ITG/anterior middle occipital gyrus, inferior occipital gyrus, and lingual gyrus, and decreased activation in R MTG and caudate nucleus.
Simos et al., 2005	MSI, Pre/Post	Letter-sound naming, pseudoword reading	Grade × Group interactions revealed reduction in onset latency in the bilateral occipito-temporal region and increased onset latency in the L IFG for responders.
Richards et al., 2006	fMRI	Orthographic mapping, Morpheme mapping with/without phonological shift, Phoneme mapping	Following intervention, the orthographic treatment group showed increased activation in R IFG and posterior parietal region to levels that no longer differed from control group.
Hoeft et al., 2007	fMRI (and VBM)	Real-word rhyme judgment	Combining fMRI and VBM with behavioral scores predicted word attack skills better than behavioral or imaging alone. Regions predicting posttest word decoding scores included R fusiform gyrus, fusiform/mid occipital gyrus, and LMTG as positive predictors and R MFG as a negative predictor.
Richards et al., 2007	fMRI, Pre/Post	Pseudoword visual decoding, aural match, and aural repeat	Following intervention, Group x Time interaction for visual-decode/aural-match contrast showed nonphonological group increased activation in L occipital cortex (BA 19) to the level of CT, whereas phonological group continued to underactivate. The aural-repeat/aural-match contrast revealed decreased activation for the phonological group to levels resembling CT in L SMG and postcentral gyrus
Simos, Fletcher, Sarkari, Billingsley-Marshall et al., 2007	MEG, Pre/Mid/Post	Timed reading of increasingly difficult words	Changes included increased degree of activity in bilateral posterior MTG (BA 21), decreased onset latency in LMTG (BA 21) and R lateral occipitotemporal region (BA 19/37), and increased onset latency in premotor cortex.
Simos, Fletcher, Sarkari, Billingsley, et al., 2007	MEG, Pre/Mid/Post	3-letter pronounceable nonwords (visually presented)	No notable activation differences between responders and nonresponders at baseline. Following intervention, responders showed increased duration of activity in the L posterior STG, SMG, and angular gyrus. The nonresponders showed increased duration of activity in R temporoparietal and bilateral frontal areas. Responders showed changes in the sequence of activation to more closely resemble CT by initiating in extrastriate, followed by temporoparietal, and then frontal areas and this temporal profile was not apparent in nonresponders.
Meyler et al., 2008	fMRI, Pre/Post/1yr Followup	Visual presentation of sentences with sense-nonsense judgment	Pre-intervention, RD underactivated in L mid occipital/angular, IPL/postcentral, SPL/sup occipital, MFG, R IPL/SMG, SMG/IPL and overactivated in anterior and posterior SMA. Post-treatment, RD activated more than CT in L putamen and R insula/IFG and CT were greater than RD in L SPL/superior occipital and MFG. At follow-up the treatment group showed greater activation than CT in L postcentral gyrus, insula/putamen, insula, SFG/cingulate, anterior SFG, anterior and middle cingulate, thalamus, and cerebellum (vermis), R postcentral gyrus, putamen/insula, SFG/SMA, anterior cingulate, posterior cingulate, precuneus, and cerebellum (vermis).
Odegard et al., 2008	fMRI, Post	Phoneme-grapheme matching, tone-symbol	Following treatment, L inferior parietal showed increased activation in controls relative to non-responders, R inferior frontal showed greater activation in responders relative to non-responders and controls, R middle temporal showed greater activation in non-responders relative to responders and controls
Richards & Berninger, 2008	fMRI, Pre/Post	Phoneme Mapping	Before treatment, children with dyslexia showed higher functional connectivity than controls from L IFG to bilateral MFG and SMA, L precentral gyrus, and R SFG. Following treatment, RD showed no difference from controls in L IFG seed point.
Davis et al., 2011	fMRI, Post	Letter-sound matching	Responders showed greater activation in the L STG (BA 22) relative to nonresponders. Responders activated more that controls in L MTG/Angular (BA39)
Farris et al., 2011	fMRI, Post	Phoneme-grapheme matching, tone-symbol	Following treatment, responders were equivalent to controls in functional connectivity between L and R inferior frontal lobes, and nonresponders exhibited less functional connectivity.
Hoeft et al., 2011	fMRI (and DTI), Pre	Rhyme judgment	fMRI activity in the R IFG (BA 44, inferior operculum) together with DTI of the R superior longitudinal fasciculus predicted responsiveness with 72% accuracy. Whole-brain multivariate patterns of brain activation (fMRI) predicted reading gains with >90% accuracy. Areas contributing to classification with positive correlation: R IFG (operculum), insula, lingual gyrus, precuneus/MTG/occipital, culmen of cerebellum, L IFG (triangularis), SFG, MFG. Negative correlation: L IFG/Insula, precentral gyrus, SFG/SMA, IPL, posterior cingulate/cuneus/calcarine, L superior/middle occipital gyri, L midbrain, R lingual/fusiform
Rezaie et al., 2011a	MEG, Pre	Word reading	At baseline, adequate responders showed increased activity in the L MTG, L STG, L ventral occipitotemporal regions, and R medial temporal cortex relative to inadequate responders. Activity in these regions predicted improvement in real word reading efficiency above predictions of reading accuracy or fluency.
Rezaie et al., 2011b	MEG, Pre	3-letter pronounceable nonwords	Pre-intervention activity was higher for adequate responders compared to inadequate responders in L SMG and angular gyrus and bilateral STG and MTG. Pre-intervention activity in L SMG, STG, and angular gyrus was positively correlated with post-intervention gains in fluency scores.
Yamada et al., 2011	fMRI, Pre/Post	One-back task with letters and letter-like stimuli	Pre-treatment at-risk group underactivated in L ITG, superior lateral occipital cortex, and thalamus, R SFG, anterior cingulate, posterior superior STG, and temporal/fusiform cortex, occipital pole, and amygdala, bilateral IFG, frontal orbital cortex (ORB), MTG, SMG, precentral cortex, SPL, supracalcarine cortex, and putamen. The at-risk group overactivated in R frontal orbital cortex (medial to the underactivation listed above). Posttreatment the at-risk group overactivated in L IFG, frontal pole, SPL, and occipital pole, R SFG, SMG, ACC, MFG, planum temporale, frontal operculum, precuneus, postcentral gyrus, lateral occipital cortex, and lingual gyrus and bilateral precentral gyrus and paracingulate region and underactivated in L superior lateral occipital cortex.
Gebauer, Fink, Kargl et al., 2012	fMRI, Pre/Post	Correctly spelled words, misspelled words, pseudowords	Treatment group showed increased activation following treatment in R posterior cingulate, L MTG, ITG, hippocampus, and parahippocampal region during pseudoword reading. The waiting group showed increases in R lateral occipital cortex and middle temporal cortex during all three conditions. CT showed increases in bilateral middle temporal and occipito-temporal regions. Group × Session interaction revealed increased activation for the training group in the bilateral parahippocampal area and cerebellum. The waiting group showed increased activation in bilateral precuneus and cerebellum, L frontal pole, and R lateral occipital cortex and parieto-temporal region.
Bach et al., in press	fMRI (and ERP), at Post-training used for predicting reading 2 years later	Word/symbol processing	fMRI and ERP data combined with behavioral measures at kindergarten significantly improved prediction of reading skill at second grade over behavioral measures alone. For fMRI, activity in L visual word form area (fusiform) ROI correlated with gains in letter knowledge.

*Note.* The terms *overactivated* and *underactivated* are used in reference to control groups. MSI  =  magnetic source imaging which combines MEG with MRI, ERP =  event related potential determined with electroencephalography, IFG  =  inferior frontal gyrus, MFG  =  middle frontal gyrus, SFG  =  superior frontal gyrus, STG  =  superior temporal gyrus, STS  =  superior temporal sulcus, MTG  =  middle temporal gyrus, ITG  =  inferior temporal gyrus, SMG  =  supramarginal gyrus, IPL  =  inferior parietal lobule, SPL  =  superior parietal lobule, SMA  =  supplementary motor area, ACC  =  anterior cingulate cortex.

### Descriptive Findings

Numerous brain regions were found to be associated with reading intervention in these studies. These regions included not only frontal, temporo-parietal, and occipital cortex, but also sublobar and subcortical areas. Below we describe the findings involved in each region in depth.

#### IFG

The IFG was found to be associated with intervention across multiple studies. Following reading intervention, previously underactivating L IFG in RD more closely resembled controls [98], [102], [105] with one study showing underactivation of at-risk children shifting to overactivation following intervention [100]. Interestingly, Richards & Berninger [106] found that children with RD showed higher functional connectivity than controls for the L IFG as related to R and L supplemental motor areas and L precentral gyrus as well as R superior frontal gyrus. Following intervention, no difference was observed between children with RD and controls. An additional functional connectivity study [107] similarly found that L and R inferior frontal connectivity was the same following treatment for RD as compared to controls, but in somewhat of a contrast, found that nonresponders to intervention showed less functional connectivity than did responders. Hoeft et al [108] found that activity in the pars triangularis of the L IFG was positively correlated with reading gains, whereas L IFG/Insula activity was negatively correlated. To summarize, it seems that L IFG involvement may be that of underactivation prior to intervention relative to controls, followed by normalization after treatment; however, there is not complete consensus among the studies in this review.

The right IFG is also prominent in findings related to intervention. Prior to treatment, at-risk children showed underactivation in R IFG [100]. Activation increases in R IFG were seen following intervention [63], [69], [98] and at follow-up [102] and considered to be normalized to the level of controls [99]. Prior to treatment, higher R IFG activity predicted higher reading gains for children with RD [108]. Following treatment, responders exhibited greater R IFG activation than did nonresponders and controls [103]. Nonresponders showed increased duration of activity in bilateral frontal areas [66], and exhibited less functional connectivity between left and right inferior frontal regions than did responders and controls [107].

#### Additional frontal areas

While the IFG was the most consistently involved region in intervention, other frontal regions emerged in some studies with several studies presenting results in superior frontal (SFG) and middle frontal (MFG) gyri. Prior to intervention, children with RD showed underactivation in R SFG [105] and increases in activation were seen following treatment [98] and at follow-up [69]. Activity in the L SFG was positively correlated in predicting response to intervention [108]. At follow-up to intervention, children with RD showed increased activation in L SFG/cingulate [69]. Prior to intervention, children with RD showed underactivation in L MFG [105] and L MFG activity contributed to whole-brain multivariate patterns that predicted responsiveness to intervention [108]. Pre-treatment activation levels in R MFG negatively correlated with posttest decoding scores [109]. Following intervention, children with RD exhibited increased levels of activation in R MFG [98], [100] whereas adults showed increased activation in R and L MFG [63]. More isolated frontal lobe findings include at-risk children underactivating in bilateral frontal orbital cortex at pre-treatment [100] and responders showing increased dorsolateral prefrontal activation[110].

#### STG and MTG

Some consistencies emerged among studies in temporo-parietal areas, particularly in the STG and MTG. Before treatment, participants with RD showed underactivation relative to controls in posterior STG and temporo-parietal cortex that increased or normalized to the level of controls after intervention [98], [100], [102], [111]. Increased L STG activation was evident at follow-up as well [100], [111]. Responders to intervention showed greater activation than nonresponders in L STG prior to treatment [112], [113] and following treatment [114]. Also, responders to treatment showed increased duration of activity in posterior L STG and the L hemisphere sequence of activation changed for responders such that temporoparietal areas activated prior to frontal areas, such that after treatment responders much more closely resembled [66]. In young children considered at-risk for RD, underactivation was seen in the R posterior STG prior to treatment. In adults, increases in R posterior STG/AG activation were seen following treatment [63]. While in one MEG study, responders exhibited bilateral temporal-parietal activation [110], another study showed nonresponders having increased R temporo-parietal activation [66]. Because of the lesser spatial resolution of MEG, these areas are more general than in fMRI.

Though the STG seemed to be the temporo-parietal region with the most consistent findings across studies, the MTG also emerged in findings from several studies. At pre-treatment, relative underactivation of the L MTG was seen, with activation increases observed following treatment [101], [102], [105], [115]. As for responsiveness, higher L MTG activity was predictive of better response to intervention [109], [112] and responders activated more than controls in L MTG/AG following intervention [114]. In addition to the L MTG, increases in activation were also shown for RD in the R MTG following treatment [98], [115]. However, Shaywitz et al [102] found that R MTG activity was higher in RD at pre-treatment than at follow-up and Odegard et al [103] found that after treatment non-responders showed greater activation relative to controls and responders in R MTG. In addition, one study found that responders had increased activity at baseline in R mesial temporal cortex, an area not identified in other studies [112].

#### Other temporo-parietal areas

Though less consistent across studies than the STG and MTG findings, activity in other temporo-parietal areas, including inferior temporal gyrus (ITG), inferior parietal lobule, SMG, and AG, was associated with reading intervention in a few studies. In the ITG, children with RD underactivated in the L hemisphere relative to controls at baseline, a difference that was no longer present following treatment [105], a finding in congruence with Gebauer, Fink, Kargl et al [101] that showed increased activation in L ITG for poor spellers/readers at post-intervention. In adults, increases in activation were seen following treatment in R and L inferior parietal lobule and left intraparietal sulcus [63]. At pretreatment, at-risk children underactivated in bilateral SMG, and following treatment activated more than controls in R SMG [100]. Pre intervention L SMG and angular gyrus activity positively correlated with post-intervention fluency gains [113]. Following intervention, responders showed increased activation of L SMG [66] and activated more than controls in L MTG/AG (BA 39). Adults showed increased activity following intervention in R STG/AG (BA 22/39) [63].

#### Occipital and fusiform

Children with RD underactivated in the occipital/fusiform region [105] and superior lateral occipital cortex [100] prior to intervention. Baseline activation in the R fusiform and R fusiform/mid occipital gyri positively correlated with later decoding scores [109]. At baseline, children who were responders to intervention showed greater activation than non-responders in L ventral occipitotemporal region [112]. Post treatment L fusiform activation positively correlated with future gains in letter knowledge for young children [116]. Following intervention, increases in activation were seen in adults in the L fusiform gyrus [63] and in children in the L lingual gyrus [98]. Onset latency increased in R lateral occipitotemporal cortex [115]. Of interest, the only region in which children who were on track for typical reading achievement activated more than at-risk children who had received treatment was in the L superior lateral occipital cortex [100]. At a one-year follow-up, increased activation was seen in occipitotemporal regions for children with RD [102].

#### Pre/postcentral

Limited evidence indicates possible differences in activity in precentral and postcentral gyri associated with intervention. Following treatment, children considered at-risk for RD showed increased activity relative to controls in L precentral gyrus [100]. Responders exhibited increased onset latency [115] of premotor cortex. Increases were seen in R inferior postcentral gyrus (BA 43) in adults following intervention [63] and in bilateral postcentral gyrus in children at follow-up [69].

#### Subcortical

Post-intervention increases in activation were found in adults with RD in the L hippocampal gyrus [63]. Increases in thalamus activity were observed in the left hemisphere following intervention for adults [63], bilaterally for children following intervention [98], and in L hemisphere for children at follow-up [69]. Following treatment, children with RD showed increased activation in L putamen relative to controls [69]. At follow-up in the same study increased activation was seen in L and R insula/putamen.

#### Insula

Following treatment, increased activation was seen in R insula/IFG [69], [98] and at follow-up in bilateral insula/putamen [69]. Prior to intervention, bilateral insular activity contributed to predicting response to intervention with a positive correlation for R insula and a negative correlation for L insula [108].

#### Additional sublobar/medial areas

Prior to intervention, the R precuneus contributed to prediction of responsiveness [108]. At treatment follow-up, children with RD showed greater activation than controls in R precuneus [69]. Children with RD showed greater activation than controls in R anterior cingulate after treatment [100] and at follow-up [69]. Prior to treatment, L posterior cingulate activation was negatively correlated in whole-brain multivariate patterns predicting responsiveness to intervention [108]. At post-intervention, poor spellers/readers showed increased activation in R posterior cingulate cortex when compared with a no-treatment group [101]. At treatment follow-up, children with RD showed greater activation than controls in R posterior cingulate [69].

### Results of ALE meta-analysis

**Figure 2:**
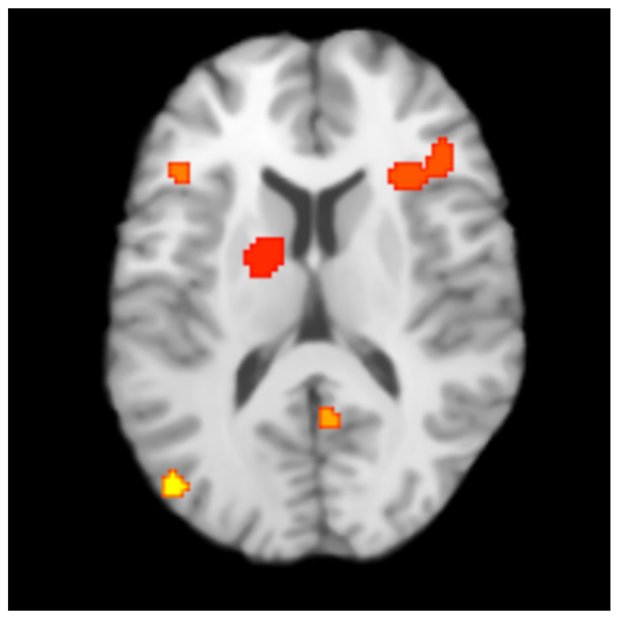
ALE results. Clusters exceeding the recommended 264^2^ threshold are L thalamus, R insula/inferior frontal gyrus, L inferior frontal gyrus, R posterior cingulate, and L middle occipital gyrus (FDR, *q*<.05; Talairach, *z* = 12).

Of the 22 studies in the qualitative literature review, eight provided sufficient information (i.e., coordinates for cluster maxima of activations at posttest when available or at follow-up when posttest was not available) to be included in ALE meta-analysis (Table 3). Results from the meta-analysis are presented in Table 4 and Fig 2. ALE revealed five clusters when using FDR of *q* = 0.05 and a cluster threshold of 264 mm^3^, the experimentally determined recommended threshold. These clusters were located in L thalamus, R Insula/IFG, L IFG, R posterior cingulate, and L middle occipital gyrus.

**Table 3:** Contrasts Used in fMRI Analyses of Studies Included in ALE.

Study	*n*	# foci	Group or Session Contrast	Task Contrast
Temple, 2003	32	14	Post > Pre for RD > CT (masked out CT clusters)	Rhyme Letters > Match Letters
Eden, 2004	38	14	Post > Pre for Intervention > No Intervention	Sound Deletion > Word Repetition
Shaywitz, 2004	25	6	Follow-up > Pre in RD	Letter/Sound Match > Tone/Symbol
Richards, 2006	8	5	Post > Pre in Orthographic group	Real Words > Letter Strings
Odegard, 2008	18	1	Post, Responder > Nonresponder & Control	Phoneme/Grapheme > Tone/Symbol
Meyler, 2008	35	3	Post, RD > CT	Sentences > Baseline Fixation
Yamada, 2011	7	40	Post > Pre in RD	Letter > False Font
Gebauer, 2012	10	7	Post > Pre in Training Group	Pseudoword >Baseline Fixation
Total	173	90		

**Table 4:** Results of ALE meta-analysis of reading intervention.

		Range (Talairach)			Local Maxima (Talairach)				
Region	Vol (mm^3^)	*x*	*y*	*z*	*x*	*y*	*z*	BA	# Studies
L Thalamus	1200	−20/−10	−14/0	4/16	−15	−6	10		3
R Insula/IFG	896	26/44	18/32	8/16					4
Insula					30	22	12	13	
IFG					42	28	12	46	
L IFG	432	−46/−38	18/24	10/20	−42	22	14	45	3
R Post Cingulate	400	0/8	−58/−52	10/20	4	−55	15	23	2
L Mid Occipital	320	−46/−40	−80/−74	8/16	−44	−78	12	19	2

Note: One additional cluster was excluded from the table because the contributing foci were from a single study.

## Discussion

### Descriptive Review

Though relatively few studies have examined the neurobiology associated with reading intervention and differing methodologies have been employed, some commonalities among studies have emerged in this review. Our descriptive literature review revealed that across studies, reading intervention may be associated with differential activation in bilateral IFG, STG, MTG, MFG, and SFG, as well as bilateral occipital regions, postcentral gyri, inferior parietal lobule, and insulae, among others. In almost all of these regions, differences involve underactivation for RD prior to intervention with relative increase following intervention. These results seem to be consistent with literature that describes underactivation in these areas for RD relative to typical readers and points toward normalization through remediation.

Not all of the patterns that emerged across these intervention studies were consistent with previous literature addressing imaging of RD. Several studies in this review indicated underactivation of L IFG prior to intervention for RD. Interestingly, while these findings are similar to that revealed in meta-analyses of RD [62] they are in contrast to the accepted model of L hemisphere dysfunction that includes L IFG overactivation in RD as a compensatory mechanism for underactivating temporo-parietal and occipito-temporal regions [41], [42] In the studies we reviewed, reading intervention was generally associated with increased L IFG activation to normalizing levels. However, as one study [100] showed overactivation in the L IFG (among other areas) in RD following intervention, there may be support for the conventional model of RD overactivating L IFG subsequent to intervention. One proposed hypothesis that would account for this pattern is that of an inverted U activation curve [68]. In this model, children with RD may have a different starting point on the curve when performing a reading task. They may start at (and remain longer) at the low point of activation. With intervention, they may exhibit increased activation in relevant areas. In contrast, unimpaired readers may start out with higher activation, but over repeated exposures or training will decrease in activation. While still speculative, the inverted U should be considered when conducting imaging studies with RD, even if the study is not directly concerned with intervention as perhaps undocumented or inadvertent treatment could be underlying increased activity.

#### Responsiveness to intervention

In addition to exploring the differences in activity associated with intervention, we were interested in finding predictors of intervention response and differences among responders and nonresponders. Individuals may vary in their neurobiological receptiveness to training or instruction. Exploring these differences may eventually facilitate the targeting of intervention to individual needs. A few studies have begun the investigation of neural predictors of reading improvement in children with RD. Pre-intervention fMRI activity in the R IFG [108] L MTG, L STG, L ventral occipitotemporal, and MEG activity in the R medial temporal cortex [112] predicts response to intervention. Hoeft et al [108] found that brain measures were more predictive of reading gains for adolescents with dyslexia than were behavioral measures, with greater activation in the R IFG during rhyme-judgment predicting greater reading improvement over the next 2 ½ years. Another study of adolescents who struggled with reading showed that brain activity prior to intervention was predictive of behavioral response to a year-long intervention that emphasized vocabulary, comprehension, word study, and fluency. Higher MEG activity in L middle, superior temporal, ventral occipitotemporal regions,and R medial temporal cortex predicted better response as demonstrated by word reading efficiency than was predicted by pre-intervention reading accuracy or fluency measures alone [112]. These areas overlap with the previously discussed posterior areas involved in reading. In general, this suggests that adolescents with neurobiological profiles more closely resembling typically achieving readers are more likely to respond well to intervention.

The studies which looked at differential responsiveness and post-intervention imaging indicated that children who demonstrate behavioral response as evidenced by standardized test scores, exhibited more activation in left middle temporal and posterior superior temporal areas [66], [114] and left inferior parietal region [103] following intervention as compared to children who did not respond to intervention. Nonresponders (a year after intervention) had increased right middle temporal lobe during a letter-sound task [103]. Functional connectivity data indicate that responders and non-impaired readers exhibit connectivity between inferior frontal regions that is absent in nonresponders [107]. Identifying these patterns of responders and nonresponders may provide insight into determining if these children are somehow at an optimal state to grow or unknowingly primed to respond to intervention, so that the lesser responders can be moved into that zone.

#### Changes: Normalizing or compensatory

Given that change in functional activity appears to be associated with reading intervention leads to consideration of what the quality or type of change is. That is, does reading intervention lead to readers with RD becoming more like typical readers or does intervention lead to compensatory changes that enhance reading ability? While the limited studies available may not present a definitive answer, they do provide some insight. Several of the studies reviewed provide evidence of normalizing changes [63], [66], [69], [98], [99], [102], [106], [115], including normalized functional connectivity [106]. However, a few studies showed evidence of possible compensatory mechanisms [63], [66], [98]. Interestingly, one study presented evidence that responders to intervention exhibit normalization while nonresponders exhibit compensation [66]. Though the evidence is limited, this may be indicative of differing types or severities of RD (i.e., less severe RD responds better both behaviorally and neurologically to intervention). Alternatively, it is possible that the responders are typical readers who have not previously received adequate instruction and when provided with adequate instruction show the functional activation profile of typical readers. Another study [63] showed adults exhibiting both normalization and compensation, illustrating that not only can adults with RD be remediated but also that the capacity for both normalizing and compensating mechanisms remains intact into adulthood.

### ALE of Post-Intervention fMRI

The narrative review of studies provides valuable information. However, synthesizing across studies is challenging when so many brain regions are involved and results are descriptive rather than quantitative. Precision may be gained by using specified coordinates of cluster activation maxima in standardized space which can then be included in meta-analysis. Meta-analysis of eight studies experiments with post-intervention data given in specified maxima coordinates statistically overlapped with the findings summarized in the narrative review; however, some of the regions that appeared most prominent in the qualitative review were not the ones revealed in the ALE analysis.

The meta-analysis showed that following intervention, participants with RD exhibited increased activation in L thalamus, R insula/IFG, L IFG, R posterior cingulate, and L middle occipital gyrus. These regions have been found to be active in processes that would presumably enhance reading ability. The L IFG likely serves multiple roles including phonologic processing and perhaps articulatory recoding [41], [42], [49]. The right IFG may play a role in attention and detection of important task related cues [117]. The finding of increased R insula activity may relate to increased coordinated relay of information and heightened detection of salient events [118], useful processes in developing the skill of reading. The role of the L thalamus appears to be complex, but activity may be indicative of the role of the left thalamus in language and verbal memory [119]. While the posterior cingulate is believed to be involved in the default mode network, a role that does not particularly coalesce with these results, the posterior cingulate also appears to be involved in memory, language, and as a hub for information exchange [120], [121].

While all the regions of the ALE results could be contextualized within the qualitative review (i.e., they overlapped with those reported to show increased activation following intervention), the regions that were statistically significant by the ALE analyses did not include some of the primary ones that are generally discussed in the literature (e.g.,L MTG and L STG). There are two possible explanations for this. On the one hand, it may simply be a result of bias due to small sample size. The meta-analysis only examined functional differences following intervention and was limited to studies that reported coordinates of activation. Because this produced a restricted subset of the studies that were in the literature review, the resulting areas that emerged are unavoidably biased and differed somewhat from the regions that predominated in the descriptive review. Alternatively, these findings could reflect the salient features of growth; that is, while the other regions may also play a role, those found in the ALE results may be the most critical regions that distinguish growth. For example, the thalamic findings are especially intriguing given the thalamus' fundamental role across many types of cognitive processes. As such, it may be a strong predictor of those who are able to compensate through other mechanisms or pathways.

### Limitations

This study has its limitations. First among these is the limited number of studies that have explored neuroimaging and reading intervention. Even within these studies there are shared participants (i.e., appears to be identical groups or overlap among studies as noted in Table 1), such that this review is fairly limited in the number of unique participants. Also, due to the limited number of studies, we included studies that differed in several respects. For instance, we included one study that was performed with adult participants, though it has been established that adults and children differ in reading activation in both typical readers and those with RD [54], [56], [73]. In addition, we included studies that greatly differed in treatment type and treatment dosage. In a study with typical adults, Sandak, Mencl, Frost, Rueckl et al [122] showed that differences in treatment affect brain activation. Clearly, if differences in functional activity are related to treatment, type and dosage of treatment would be important factors. Additionally, though our focus was on RD, there is not a single definition for RD or being at-risk for RD and we found that the definition of RD varied by study.

A further limitation of the study is that neither the narrative review nor the ALE meta-analysis considered the statistical thresholds for imaging analysis used by each experiment. Not all studies approach data analysis in the same way, which presents a concern when synthesizing across studies. An experiment with more liberal thresholds of significance or cluster size would have its coordinates entered into the ALE meta-analysis with equal weight to that of an experiment (of comparable size) that used more stringent thresholds. In some instances, journal articles do not provide all details of their methods and analysis. In more recent years, a call for more explicit description of experiment methodology and data analysis has occurred [123]. As the functional imaging field has grown and evolved, reporting of methods and results have generally improved with the understanding of what information is necessary to the reader. This is a natural progression for a field in its infancy, so it is with this perspective that we acknowledge that shortcomings in earlier works were likely due to limitations of the time. Nonetheless, it is important to state that at least one study appears to have used an uncorrected significance threshold and in several of the studies the reporting of methodology and statistical analysis is somewhat opaque. In one respect, our analyses are limited by the analyses performed in the original studies. In another respect, meta-analytic techniques are useful in identifying commonalities across studies and could add validity to studies that did not use stringent statistics if the results are consistent across studies.

Another limitation of this study is that we did not address task difficulty in our analyses. Task difficulty may have an effect on functional activity and this effect may differ depending on reading proficiency. As Pugh et al [68] showed, factors that make words easier to read (i.e., frequency, imageability, consistency) result in higher activation in reading related brain areas for RD while non-impaired readers exhibit, reduced activation. Interestingly, there is indication that even in a resting state condition, proficient readers show activation in reading networks [124]. This is an area of research that needs further exploration.

Another important consideration is we did not directly examine age-related differences. Gray matter development is not uniform. Rather, while areas involved in basic function such as motor and sensory areas mature early, areas involved in executive function, attention, and motor coordination mature later, with areas involving spatial orientation, speech and language development maturing in-between. As stated previously, functional changes occur over the course of development as well [55]. As such, when considering activity in a certain region of the brain, it is worthwhile to consider the typical course of functional activity in that region.

### Conclusion and Future Directions

Despite the limited number of studies and the disparate methods of experiments included due to this low number, this analysis is a start at examining reading intervention and neuroimaging not only in a broad descriptive sense, but also in a quantitative manner. While acknowledging the limitations, the current results suggest that there are differences in brain activity associated with intervention. More studies using neuroimaging to explore reading intervention are needed to gain fuller understanding of functional activity and its relation to treatment. As reporting of functional data increasingly includes coordinates of cluster maxima, future meta-analyses will be able to incorporate more experiments. Also, we did not explore differences in type of intervention or dosages with regard to corresponding imaging data. Recently, an fMRI study of novel pseudoword learning indicated that the type of training used in new word learning may matter more to less skilled readers than to highly skilled readers [125], so there is certainly a need to explore this in future analyses. As neuroimaging studies become more numerous and investigators become more adept at analyzing and reporting the data in sufficient detail, more targeted meta-analyses can be conducted.

This paper is an effort to review functional imaging findings that are associated with reading intervention. Using neuroimaging to explore intervention is not a new concept [126], yet because these studies are complex, expensive, and time consuming, relatively few have been conducted. This is an early examination of an area of research that has substantial room to grow. Accordingly, caution should be used in interpreting results. Few conclusions can be drawn at this early stage, but this is an effort to guide future research and to underscore the need for larger scale studies. At this point, we are approaching this as a basic science question, seeking to better define one piece of a very complex puzzle. It is far too soon to suggest any practical implications, yet future studies may lead to practical applications that eventually impact treatment of RD.

## Supporting Information

Checklist S1 **PRISMA checklist.**
(DOC)

## References

[1] Stanovich KE (1986) Matthew effects in reading: Some consequences of individual differences in the acquisition of literacy. Read Res Q 21: 360–407.

[2] Morgan PL, Farkas G, Hibel J (2008) Matthew effects for whom? Learn Disabil Q 31: 187–198. 26339117 PMC4554759

[3] Fletcher JM (2009) Dyslexia: The evolution of a scientific concept. J Int Neuropsychol Soc 15: 501–508 doi:10.1017/S1355617709090900 19573267 10.1017/S1355617709090900 PMC3079378

[4] Torgesen JK, Wagner RK, Rashotte CA, Rose E, Lindamood P, et al (1999) Preventing reading failure in young children with phonological processing disabilities: Group and individual responses to instruction. J Educ Psychol 91: 579–593.

[5] Shankweiler D, Lundquist E, Katz L, Stuebing KK, Fletcher JM, et al (1999) Comprehension and Decoding: Patterns of Association in Children With Reading Difficulties. Sci Stud Read 3: 69–94 doi:_10.1207/s1532799xssr0301_4

[6] Nation K, Snowling M (1997) Assessing reading difficulties: The validity and utility of current measures of reading skill. Br J Educ Psychol 67: 359–370. 9376312 10.1111/j.2044-8279.1997.tb01250.x

[7] Catts H, Compton DL, Tomblin JB, Bridges MS (2012) Prevalence and nature of late-emerging poor readers. J Educ Psychol 104: 166–181. 10.1037/a0025323 PMC3835401 24273341

[8] Compton DL, Fuchs D, Fuchs LS, Elleman AM, Gilbert JK (2008) Tracking children who fly below the radar: Latent transition modeling of students with late-emerging reading disability. Learn Individ Differ 18: 329–337 doi:10.1016/j.lindif.2008.04.003

[9] Leach JM, Scarborough HS, Rescorla L (2003) Late-emerging reading disabilities. J Educ Psychol 95: 211–224 doi:10.1037/0022-0663.95.2.211

[10] Lipka O, Lesaux NK, Siegel LS (2006) Retrospective Analyses of the Reading Development of Grade 4 Students with Reading Disabilities: Risk Status and Profiles Over 5 Years. J Learn Disabil 39: 364–378 doi:10.1177/00222194060390040901 16895160 10.1177/00222194060390040901

[11] Torgesen JK (2000) Individual differences in response to early interventions in reading: The lingering problem of treatment resisters. Learn Disabil Res Pract 15: 55–64.

[12] Al Otaiba S, Fuchs D (2002) Characteristics of Children Who Are Unresponsive to Early Literacy Intervention: A Review of the Literature. Remedial Spec Educ 23: 300–316 doi:10.1177/07419325020230050501

[13] Foorman BR, Francis DJ, Fletcher JM, Schatschneider C, Mehta P (1998) The role of instruction in learning to read: Preventing reading failure in at-risk children. J Educ Psychol 90: 37–55 doi:10.1037//0022-0663.90.1.37

[14] Vellutino FR, Scanlon DM, Sipay ER, Small SG, Pratt A, et al (1996) Cognitive profiles of difficult-to-remediate and readily remediated poor readers: Early intervention as a vehicle for distinguishing between cognitive and experiential deficits as basic causes of specific reading disability. J Educ Psychol 88: 601–638.

[15] Vaughn S, Wanzek J, Wexler J, Barth A, Cirino PT, et al (2009) The relative effects of group size on reading progress of older students with reading difficulties. Read Writ 23: 931–956 doi:10.1007/s11145-009-9183-9 10.1007/s11145-009-9183-9 PMC2975110 21072131

[16] Compton DL, Gilbert JK, Jenkins JR, Fuchs D, Fuchs LS, et al (2012) Accelerating chronically unresponsive children to tier 3 instruction: what level of data is necessary to ensure selection accuracy? J Learn Disabil 45: 204–216 doi:10.1177/0022219412442151 22491810 10.1177/0022219412442151

[17] National Reading Panel (2000) Teaching children to read: An evidence-based assessment of the scientific research literature on reading and its implications for reading instruction. Washington, DC: National Institues of Health and Human Development.

[18] Vaughn S, Gersten R, Chard DJ (2000) The underlying message in LD intervention research: Findings from research syntheses. Except Child 67..

[19] Swanson HL (1999) Reading Research for Students with LD: A Meta-Analysis of Intervention Outcomes. J Learn Disabil 32: 504–532 doi:10.1177/002221949903200605 15510440 10.1177/002221949903200605

[20] Bus AG, van IJzendoorn MH (1999) Phonological awareness and early reading: A meta-analysis of experimental training studies. J Educ Psychol 91: 403–414.

[21] Nelson JR, Benner GJ, Gonzalez J (2003) Learner characteristics that influence the treatment effectiveness of early literacy interventions: A meta-analytic review. Learn Disabil Res Pract 18: 255–267.

[22] Tran L, Sanchez T, Arellano B, Swanson HL (2011) A meta-analysis of the RTI literature for children at risk for reading disabilities. J Learn Disabil 44: 283–295 doi:10.1177/0022219410378447 21521870 10.1177/0022219410378447

[23] Frith U (2001) What framework should we use for understanding developmental disorders? Dev Neuropsychol 20: 555–563 doi:_10.1207/S15326942DN2002_6 11892952 10.1207/S15326942DN2002_6

[24] Fletcher JM, Lyon GR (2008) Dyslexia: Why precise definitions are important and how we have achieved them. Perspect Lang Lit 34: 27–31.

[25] Vellutino FR, Fletcher JM, Snowling MJ, Scanlon DM (2004) Specific reading disability (dyslexia): What have we learned in the past four decades? J Child Psychol Psychiatry 45: 2–40. 14959801 10.1046/j.0021-9630.2003.00305.x

[26] Leonard CM, Eckert MA (2008) Asymmetry and dyslexia. Dev Neuropsychol 33: 663–681 doi:10.1080/87565640802418597 19005910 10.1080/87565640802418597 PMC2586924

[27] Miller B, McCardle P (2011) Moving closer to a public health model of language and learning disabilities: the role of genetics and the search for etiologies. Behav Genet 41: 1–5 doi:10.1007/s10519-010-9439-9 21229298 10.1007/s10519-010-9439-9 PMC3897164

[28] Chein JM, Schneider W (2005) Neuroimaging studies of practice-related change: fMRI and meta-analytic evidence of a domain-general control network for learning. Brain Res Cogn Brain Res 25: 607–623 doi:10.1016/j.cogbrainres.2005.08.013 16242923 10.1016/j.cogbrainres.2005.08.013

[29] Brem S, Bach S, Kucian K, Guttorm TK, Martin E, et al (2010) Brain sensitivity to print emerges when children learn letter-speech sound correspondences. Proc Natl Acad Sci U S A 107: 7939–7944 doi:10.1073/pnas.0904402107 20395549 10.1073/pnas.0904402107 PMC2867899

[30] Gomez-Pinilla F (2008) Brain foods: the effects of nutrients on brain function. Nat Rev Neurosci 9: 568–578 doi:10.1038/nrn2421 18568016 10.1038/nrn2421 PMC2805706

[31] Day JJ, Sweatt JD (2011) Epigenetic mechanisms in cognition. Neuron 70: 813–829 doi:10.1016/j.neuron.2011.05.019 21658577 10.1016/j.neuron.2011.05.019 PMC3118503

[32] Sweatt JD (2009) Experience-dependent epigenetic modifications in the central nervous system. Biol Psychiatry 65: 191–197 doi:10.1016/j.biopsych.2008.09.002 19006788 10.1016/j.biopsych.2008.09.002 PMC3090137

[33] Vaynman S, Gomez-Pinilla F (2006) Revenge of the “sit”: how lifestyle impacts neuronal and cognitive health through molecular systems that interface energy metabolism with neuronal plasticity. J Neurosci Res 84: 699–715 doi:10.1002/jnr.20979 16862541 10.1002/jnr.20979

[34] Lynch MA (2004) Long-term potentiation and memory. Physiol Rev 84: 87–136. 14715912 10.1152/physrev.00014.2003

[35] Cooke SF, Bliss TV (2006) Plasticity in the human central nervous system. Brain 129: 1659–1673 doi:10.1093/brain/awl082 16672292 10.1093/brain/awl082

[36] Kandel ER (2001) The molecular biology of memory storage: a dialogue between genes and synapses. Science 294: 1030–1038 doi:10.1126/science.1067020 11691980 10.1126/science.1067020

[37] Zatorre RJ, Fields RD, Johansen-Berg H (2012) Plasticity in gray and white: neuroimaging changes in brain structure during learning. Nat Neurosci 15: 528–536 doi:10.1038/nn.3045 22426254 10.1038/nn.3045 PMC3660656

[38] Bredy TW, Lin Q, Wei W, Baker-Andresen D, Mattick JS (2011) MicroRNA regulation of neural plasticity and memory. Neurobiol Learn Mem 96: 89–94 doi:10.1016/j.nlm.2011.04.004 21524708 10.1016/j.nlm.2011.04.004

[39] Lonze BE, Ginty DD (2002) Function and Regulation of CREB Family Transcription Factors in the Nervous System CREB and its close relatives are now widely accepted. 35: 605–623. 10.1016/s0896-6273(02)00828-0 12194863

[40] Kogan JH, Frankland PW, Blendy JA, Coblentz J, Marowitz Z, et al (1996) Spaced training induces normal long-term memory in CREB mutant mice. Curr Biol 7: 1–11. 10.1016/s0960-9822(06)00022-4 8999994

[41] Pugh KR, Mencl WE, Jenner AR, Katz L, Frost SJ, et al (2000) Functional neuroimaging studies of reading and reading disability (developmental dyslexia). Ment Retard Dev Disabil Res Rev 6: 207–213. 10982498 10.1002/1098-2779(2000)6:3<207::AID-MRDD8>3.0.CO;2-P

[42] Pugh KR, Mencl WE, Jenner AR, Katz L, Frost SJ, et al (2001) Neurobiological studies of reading and reading disability. J Commun Disord 34: 479–492. 11725860 10.1016/s0021-9924(01)00060-0

[43] Schlaggar BL, McCandliss BD (2007) Development of neural systems for reading. Annu Rev Neurosci 30: 475–503 doi:10.1146/annurev.neuro.28.061604.135645 17600524 10.1146/annurev.neuro.28.061604.135645

[44] Shaywitz SE, Shaywitz BA (2008) Paying attention to reading: the neurobiology of reading and dyslexia. Dev Psychopathol 20: 1329–1349 doi:10.1017/S0954579408000631 18838044 10.1017/S0954579408000631

[45] Cohen L, Dehaene S, Chochon F, Lehericy S, Naccache L (2000) Language and calculation within the parietal lobe: a combined cognitive, anatomical and fMRI study. Neuropsychologia 38: 1426–1440. 10869586 10.1016/s0028-3932(00)00038-5

[46] McCandliss BD, Cohen L, Dehaene S (2003) The visual word form area: expertise for reading in the fusiform gyrus. Trends Cogn Sci 7: 293–299 doi:10.1016/s1364-6613(03)00134-7 12860187 10.1016/s1364-6613(03)00134-7

[47] Price CJ (2012) A review and synthesis of the first 20 years of PET and fMRI studies of heard speech, spoken language and reading. Neuroimage 62: 816–847 doi:10.1016/j.neuroimage.2012.04.062 22584224 10.1016/j.neuroimage.2012.04.062 PMC3398395

[48] Dehaene S, Cohen L (2011) The unique role of the visual word form area in reading. Trends Cogn Sci 15: 254–262 doi:10.1016/j.tics.2011.04.003 21592844 10.1016/j.tics.2011.04.003

[49] Levy J, Pernet C, Treserras S, Boulanouar K, Berry I, et al (2008) Piecemeal recruitment of left-lateralized brain areas during reading: a spatio-functional account. Neuroimage 43: 581–591 doi:10.1016/j.neuroimage.2008.08.008 18778780 10.1016/j.neuroimage.2008.08.008

[50] Temple E (2002) Brain mechanisms in normal and dyslexic readers. Curr Opin Neurobiol 12: 178–183. 12015234 10.1016/s0959-4388(02)00303-3

[51] Shaywitz SE, Shaywitz BA, Pugh KR, Fulbright RK, Constable RT, et al (1998) Functional disruption in the organization of the brain for reading in dyslexia. Proc Natl Acad Sci USA 95: 2636–2641. 9482939 10.1073/pnas.95.5.2636 PMC19444

[52] Constable RT, Pugh KR, Berroya E, Mencl WE, Westerveld M, et al (2004) Sentence complexity and input modality effects in sentence comprehension: an fMRI study. Neuroimage 22: 11–21 doi:10.1016/j.neuroimage.2004.01.001 15109993 10.1016/j.neuroimage.2004.01.001

[53] Cutting LE, Clements AM, Courtney S, Rimrodt SL, Schafer JG, et al (2006) Differential components of sentence comprehension: beyond single word reading and memory. Neuroimage 29: 429–438 doi:10.1016/j.neuroimage.2005.07.057 16253527 10.1016/j.neuroimage.2005.07.057

[54] Brown TT, Lugar HM, Coalson RS, Miezin FM, Petersen SE, et al (2005) Developmental changes in human cerebral functional organization for word generation. Cereb cortex 15: 275–290 doi:10.1093/cercor/bhh129 15297366 10.1093/cercor/bhh129

[55] Schlaggar BL, Church JA (2009) Functional Neuroimaging Insights Into the Development of Skilled Reading. Curr Dir Psychol Sci 18: 21–26 doi:10.1111/j.1467-8721.2009.01599.x 19750204 10.1111/j.1467-8721.2009.01599.x PMC2741313

[56] Church JA, Coalson RS, Lugar HM, Petersen SE, Schlaggar BL (2008) A developmental fMRI study of reading and repetition reveals changes in phonological and visual mechanisms over age. Cereb cortex 18: 2054–2065 doi:10.1093/cercor/bhm228 18245043 10.1093/cercor/bhm228 PMC2517103

[57] Ben-Shachar M, Dougherty RF, Deutsch GK, Wandell BA (2011) The development of cortical sensitivity to visual word forms. J Cogn Neurosci 23: 2387–2399. 21261451 10.1162/jocn.2011.21615 PMC3214009

[58] McCandliss BD, Noble KG (2003) The development of reading impairment: A cognitive neuroscience model. Ment Retard Dev Disabil Res Rev 9: 196–204 doi:10.1002/Mrdd.10080 12953299 10.1002/mrdd.10080

[59] Démonet J-F, Taylor MJ, Chaix Y (2004) Developmental dyslexia. Lancet 363: 1451–1460 doi:10.1016/s0140-6736(04)16106-0 15121410 10.1016/S0140-6736(04)16106-0

[60] Sandak R, Mencl WE, Frost SJ, Pugh KR (2004) The Neurobiological Basis of Skilled and Impaired Reading: Recent Findings and New Directions. Sci Stud Read 8: 273–292 doi:_10.1207/s1532799xssr0803_6

[61] Shaywitz SE, Shaywitz BA (2005) Dyslexia (specific reading disability). Biol Psychiatry 57: 1301–1309 doi:10.1016/j.biopsych.2005.01.043 15950002 10.1016/j.biopsych.2005.01.043

[62] Richlan F (2012) Developmental dyslexia: dysfunction of a left hemisphere reading network. Front Hum Neurosci 6: 120 doi:10.3389/fnhum.2012.00120 22557962 10.3389/fnhum.2012.00120 PMC3340948

[63] Eden GF, Jones KM, Cappell K, Gareau L, Wood FB, et al (2004) Neural changes following remediation in adult developmental dyslexia. Neuron 44: 411–422 doi:10.1016/j.neuron.2004.10.019 15504323 10.1016/j.neuron.2004.10.019

[64] Rimrodt SL, Clements-Stephens AM, Pugh KR, Courtney SM, Gaur P, et al (2009) Functional MRI of Sentence Comprehension in Children with Dyslexia: Beyond Word Recognition. Cereb cortex 19: 402–413 doi:10.1093/cercor/bhn092 18515796 10.1093/cercor/bhn092 PMC2638788

[65] Sarkari S, Simos PG, Fletcher JM, Castillo EM, Breier JI, et al (2002) Contributions of magnetic source imaging to the understanding of dyslexia. Semin Pediatr Neurol 9: 229–238. 12350044 10.1053/spen.2002.35506

[66] Simos PG, Fletcher JM, Sarkari S, Billingsley RL, Denton C, et al (2007) Altering the brain circuits for reading through intervention: a magnetic source imaging study. Neuropsychology 21: 485–496 doi:10.1037/0894-4105.21.4.485 17605581 10.1037/0894-4105.21.4.485

[67] Bach S, Brandeis D, Hofstetter C, Martin E, Richardson U, et al (2010) Early emergence of deviant frontal fMRI activity for phonological processes in poor beginning readers. Neuroimage 53: 682–693 doi:10.1016/j.neuroimage.2010.06.039 20600985 10.1016/j.neuroimage.2010.06.039

[68] Pugh KR, Frost SJ, Sandak R, Landi N, Rueckl JG, et al (2008) Effects of stimulus difficulty and repetition on printed word identification: An fMRI comparison of nonimpaired and reading-disabled adolescent cohorts. J Cogn Neurosci 20: 1146–1160. 18284344 10.1162/jocn.2008.20079 PMC3152957

[69] Meyler A, Keller TA, Cherkassky VL, Gabrieli JDE, Just MA (2008) Modifying the brain activation of poor readers during sentence comprehension with extended remedial instruction: A longitudinal study of neuroplasticity. Neuropsychologia 46: 2580–2592 doi:10.1016/j.neuropsychologia.2008.03.012 18495180 10.1016/j.neuropsychologia.2008.03.012 PMC2598765

[70] Little DM, Thulborn KR (2006) Prototype-distortion category learning: A two-phase learning process across a distributed network. Brain Cogn 60: 233–243 doi:10.1016/j.bandc.2005.06.004 16406637 10.1016/j.bandc.2005.06.004

[71] Maisog JM, Einbinder ER, Flowers DL, Turkeltaub PE, Eden GF (2008) A meta-analysis of functional neuroimaging studies of dyslexia. Ann N Y Acad Sci 1145: 237–259 doi:10.1196/annals.1416.024 19076401 10.1196/annals.1416.024

[72] Richlan F, Kronbichler M, Wimmer H (2009) Functional Abnormalities in the Dyslexic Brain: A Quantitative Meta-Analysis of Neuroimaging Studies. Hum Brain Mapp 30: 3299–3308 doi:10.1002/Hbm.20752 19288465 10.1002/hbm.20752 PMC2989182

[73] Richlan F, Kronbichler M, Wimmer H (2011) Meta-analyzing brain dysfunctions in dyslexic children and adults. Neuroimage 56: 1735–1742 doi:10.1016/j.neuroimage.2011.02.040 21338695 10.1016/j.neuroimage.2011.02.040

[74] Richards TL, Aylward EH, Field KM, Grimme AC, Raskind W, et al (2006) Converging evidence for triple word form theory in children with dyslexia. Dev Neuropsychol 30: 547–589 doi:_10.1207/s15326942dn3001_3 16925475 10.1207/s15326942dn3001_3

[75] Noble KG, McCandliss BD (2005) Reading development and impairment: Behavioral, social, and neurobiological factors. Dev Behav Pediatr 26: 370–378. 10.1097/00004703-200510000-00006 16222178

[76] Shaywitz BA, Lyon GR, Shaywitz SE (2006) The role of functional magnetic resonance imaging in understanding reading and dyslexia. Dev Neuropsychol 30: 613–632 doi:_10.1207/s15326942dn3001_5 16925477 10.1207/s15326942dn3001_5

[77] Shaywitz SE, Morris R, Shaywitz BA (2008) The education of dyslexic children from childhood to young adulthood. Annu Rev Psychol 59: 451–475 doi:10.1146/annurev.psych.59.103006.093633 18154503 10.1146/annurev.psych.59.103006.093633

[78] Davis N, Fan Q, Compton DL, Fuchs D, Fuchs LS, et al (2010) Influences of Neural Pathway Integrity on Children's Response to Reading Instruction. Front Syst Neurosci 4: 150 doi:10.3389/fnsys.2010.00150 21088707 10.3389/fnsys.2010.00150 PMC2982724

[79] Keller TA, Just MA (2009) Altering cortical connectivity: remediation-induced changes in the white matter of poor readers. Neuron 64: 624–631 doi:10.1016/j.neuron.2009.10.018 20005820 10.1016/j.neuron.2009.10.018 PMC2796260

[80] Gebauer D, Fink A, Filippini N, Johansen-Berg H, Reishofer G, et al.. (2011) Differences in integrity of white matter and changes with training in spelling impaired children: a diffusion tensor imaging study. Brain Struct Funct. doi:10.1007/s00429-011-0371-4 10.1007/s00429-011-0371-4 PMC3672831 22198594

[81] Krafnick AJ, Flowers DL, Napoliello EM, Eden GF (2011) Gray matter volume changes following reading intervention in dyslexic children. Neuroimage 57: 733–741 doi:10.1016/j.neuroimage.2010.10.062 21029785 10.1016/j.neuroimage.2010.10.062 PMC3073149

[82] Spironelli C, Penolazzi B, Vio C, Angrilli A (2010) Cortical reorganization in dyslexic children after phonological training: evidence from early evoked potentials. Brain 133: 3385–3395 doi:10.1093/brain/awq199 20688811 10.1093/brain/awq199

[83] Lovio R, Halttunen A, Lyytinen H, Näätänen R, Kujala T (2012) Reading skill and neural processing accuracy improvement after a 3-hour intervention in preschoolers with difficulties in reading-related skills. Brain Res 1448: 42–55 doi:10.1016/j.brainres.2012.01.071 22364735 10.1016/j.brainres.2012.01.071

[84] Richards TL, Corina D, Serafini S, Steury K, Echelard DR, et al (2000) Effects of a phonologically driven treatment for dyslexia on lactate levels measured by proton MR spectroscopic imaging. Am J Neuroradiol 21: 916–922. 10815668 PMC7976747

[85] Richards TL, Berninger VW, Aylward EH, Richards AL, Thomson JB, et al (2002) Reproducibility of proton MR spectroscopic imaging (PEPSI): comparison of dyslexic and normal-reading children and effects of treatment on brain lactate levels during language tasks. Am J Neuroradiol 23: 1678–1685. 12427623 PMC8185817

[86] Costanzo F, Menghini D, Caltagirone C, Oliveri M, Vicari S (2012) High frequency rTMS over the left parietal lobule increases non-word reading accuracy. Neuropsychologia 50: 2645–2651 doi:10.1016/j.neuropsychologia.2012.07.017 22820638 10.1016/j.neuropsychologia.2012.07.017

[87] Turkeltaub PE, Benson J, Hamilton RH, Datta A, Bikson M, et al (2012) Left lateralizing transcranial direct current stimulation improves reading efficiency. Brain Stimul 5: 201–207 doi:10.1016/j.brs.2011.04.002 22305346 10.1016/j.brs.2011.04.002 PMC3346858

[88] Turkeltaub PE, Eden GF, Jones KM, Zeffiro TA (2002) Meta-Analysis of the Functional Neuroanatomy of Single-Word Reading: Method and Validation. Neuroimage 16: 765–780 doi:10.1006/nimg.2002.1131 12169260 10.1006/nimg.2002.1131

[89] Laird AR (n.d.) GingerALE 2.1.1. Brainmap website. Available: http://www.brainmap.org. Accessed: 27 Marh 2012.

[90] Laird AR, Fox PM, Price CJ, Glahn DC, Uecker AM, et al (2005) ALE meta-analysis: controlling the false discovery rate and performing statistical contrasts. Hum Brain Mapp 25: 155–164 doi:10.1002/hbm.20136 15846811 10.1002/hbm.20136 PMC6871747

[91] Eickhoff SB, Laird AR, Grefkes C, Wang LE, Zilles K, et al (2009) Coordinate-based activation likelihood estimation meta-analysis of neuroimaging data: a random-effects approach based on empirical estimates of spatial uncertainty. Hum Brain Mapp 30: 2907–2926 doi:10.1002/hbm.20718 19172646 10.1002/hbm.20718 PMC2872071

[92] Turkeltaub PE, Eickhoff SB, Laird AR, Fox M, Wiener M, et al (2012) Minimizing within-experiment and within-group effects in Activation Likelihood Estimation meta-analyses. Hum Brain Mapp 33: 1–13 doi:10.1002/hbm.21186 21305667 10.1002/hbm.21186 PMC4791073

[93] Laird AR (2011) User Manual for GingerALE 2.1. Brainmap website. Available: http://www.brainmap.org. Accessed: 27 Mar 2012.

[94] Moher D, Liberati A, Tetzlaff J, Altman DG (2009) The PRISMA Group (2009) Preferred reporting items for systematic reviews and meta-analyses: The PRISMA statement. PLoS Med 6: 1–6 doi:10.1371/ PMC3090117 21603045

[95] Lancaster JL, Tordesillas-Gutierrez D, Martinez M, Salinas F, Evans A, et al (2007) Bias between MNI and Talairach coordinates analyzed using the ICBM-152 brain template. Hum Brain Mapp 28: 1194–1205. 17266101 10.1002/hbm.20345 PMC6871323

[96] Laird AR, Robinson JL, McMillan KM, Tordesillas-Gutierrez D, Moran ST, et al (2010) Comparison of the disparity between Talairach and MNI coordinates in functional neuroimaging data: Validation of the Lancaster transform. Neuroimage 51: 677–683. 20197097 10.1016/j.neuroimage.2010.02.048 PMC2856713

[97] Eickhoff SB, Bzdok D, Laird AR, Kurth F, Fox PT (2012) Activation likelihood estimation revisited. Neuroimage 59: 2349–2361. 21963913 10.1016/j.neuroimage.2011.09.017 PMC3254820

[98] Temple E, Deutsch GK, Poldrack RA, Miller SL, Tallal P, et al (2003) Neural deficits in children with dyslexia ameliorated by behavioral remediation: Evidence from functional MRI. Proc Natl Acad Sci U S A 100: 2860–2865 doi:10.1073/pnas.0030098100 12604786 10.1073/pnas.0030098100 PMC151431

[99] Richards TL, Aylward EH, Berninger VW, Field KM, Grimme AC, et al (2006) Individual fMRI activation in orthographic mapping and morpheme mapping after orthographic or morphological spelling treatment in child dyslexics. J Neurolinguistics 19: 56–86 doi:10.1016/j.jneuroling.2005.07.003

[100] Yamada Y, Stevens C, Dow M, Harn BA, Chard DJ, et al (2011) Emergence of the neural network for reading in five-year-old beginning readers of different levels of pre-literacy abilities: an fMRI study. Neuroimage 57: 704–713 doi:10.1016/j.neuroimage.2010.10.057 20977940 10.1016/j.neuroimage.2010.10.057 PMC3129372

[101] Gebauer D, Fink A, Kargl R, Reishofer G, Koschutnig K, et al (2012) Differences in brain function and changes with intervention in children with poor spelling and reading abilities. PLoS One 7: e38201 doi:10.1371/journal.pone.0038201 22693600 10.1371/journal.pone.0038201 PMC3364962

[102] Shaywitz BA, Shaywitz SE, Blachman BA, Pugh KR, Fulbright RK, et al (2004) Development of left occipitotemporal systems for skilled reading in children after a phonologically- based intervention. Biol Psychiatry 55: 926–933 doi:10.1016/j.biopsych.2003.12.019 15110736 10.1016/j.biopsych.2003.12.019

[103] Odegard TN, Ring J, Smith S, Biggan J, Black J (2008) Differentiating the neural response to intervention in children with developmental dyslexia. Ann Dyslexia 58: 1–14 doi:10.1007/s11881-008-0014-5 18483867 10.1007/s11881-008-0014-5

[104] Mango version 2.6 (2012). Res Imaging Inst website. Available: http://ric.uthscsa.edu/mango/. Accessed: 20 Feb 2013.

[105] Aylward EH, Richards TL, Berninger VW, Nagy WE, Field KM, et al (2003) Instructional treatment associated with changes in brain activation in children with dyslexia. Neurology 61: 212–219. 12874401 10.1212/01.wnl.0000068363.05974.64

[106] Richards TL, Berninger VW (2008) Abnormal fMRI connectivity in children with dyslexia during a phoneme task: Before but not after treatment. J Neurolinguistics 21: 294–304 doi:10.1016/j.jneuroling.2007.07.002 19079567 10.1016/j.jneuroling.2007.07.002 PMC2597820

[107] Farris EA, Odegard TN, Miller HL, Ring J, Allen G, et al (2011) Functional connectivity between the left and right inferior frontal lobes in a small sample of children with and without reading difficulties. Neurocase 17: 425–439. 21590585 10.1080/13554794.2010.532141

[108] Hoeft F, McCandliss BD, Black JM, Gantman A, Zakerani N, et al (2011) Neural systems predicting long-term outcome in dyslexia. Proc Natl Acad Sci U S A 108: 361–366 doi:10.1073/pnas.1008950108 21173250 10.1073/pnas.1008950108 PMC3017159

[109] Hoeft F, Ueno T, Reiss AL, Meyler A, Whitfield-Gabrieli S, et al (2007) Prediction of children's reading skills using behavioral, functional, and structural neuroimaging measures. Behav Neurosci 121: 602–613 doi:10.1037/0735-7044.121.3.602 17592952 10.1037/0735-7044.121.3.602

[110] Simos PG, Fletcher JM, Sarkari S, Billingsley RL, Francis DJ, et al (2005) Early development of neurophysiological processes involved in normal reading and reading disability: a magnetic source imaging study. Neuropsychology 19: 787–798 doi:10.1037/0894-4105.19.6.787 16351354 10.1037/0894-4105.19.6.787

[111] Simos PG, Fletcher JM, Bergman E, Breier JI, Foorman BR, et al (2002) Dyslexia-specific brain activation profile becomes normal following successful remedial training. Neurology 58: 1203–1213. 11971088 10.1212/wnl.58.8.1203

[112] Rezaie R, Simos PG, Fletcher JM, Cirino PT, Vaughn S, et al (2011) Engagement of temporal lobe regions predicts response to educational interventions in adolescent struggling readers. Dev Neuropsychol 36: 869–888 doi:10.1080/87565641.2011.606404 21978010 10.1080/87565641.2011.606404 PMC3308683

[113] Rezaie R, Simos PG, Fletcher JM, Cirino PT, Vaughn S, et al (2011) Temporo-parietal brain activity as a longitudinal predictor of response to educational interventions among middle school struggling readers. J Int Neuropsychol Soc 17: 875–885 doi:10.1017/S1355617711000890 21740612 10.1017/S1355617711000890 PMC3174865

[114] Davis N, Barquero L, Compton DL, Fuchs LS, Fuchs D, et al (2011) Functional correlates of children's responsiveness to intervention. Dev Neuropsychol 36: 288–301. 21462008 10.1080/87565641.2010.549875 PMC4416061

[115] Simos PG, Fletcher JM, Sarkari S, Billingsley-Marshall R, Denton CA, et al (2007) Intensive instruction affects brain magnetic activity associated with oral word reading in children with persistent reading disabilities. J Learn Disabil 40: 37–48. 17274546 10.1177/00222194070400010301

[116] Bach S, Richardson U, Brandeis D, Martin E, Brem S (n.d.) Print-specific multimodal brain activation in kindergarten improves prediction of reading skills in second grade. Neuroimage. doi:10.1016/j.neuroimage.2011.07.023 10.1016/j.neuroimage.2013.05.062 23727320

[117] Hampshire A, Chamberlain SR, Monti MM, Duncan J, Owen AM (2010) The role of the right inferior frontal gyrus: inhibition and attentional control. Neuroimage 50: 1313–1319 doi:10.1016/j.neuroimage.2009.12.109 20056157 10.1016/j.neuroimage.2009.12.109 PMC2845804

[118] Menon V, Uddin LQ (2010) Saliency, switching, attention and control: a network model of insula function. Brain Struct Funct 214: 655–667 doi:10.1007/s00429-010-0262-0 20512370 10.1007/s00429-010-0262-0 PMC2899886

[119] Hebb AO, Ojemann GA (2012) The thalamus and language revisited. Brain Lang. doi:10.1016/j.bandl.2012.06.010 10.1016/j.bandl.2012.06.010 22857902

[120] Leech R, Braga R, Sharp DJ (2012) Echoes of the brain within the posterior cingulate cortex. J Neurosci 32: 215–222 doi:10.1523/JNEUROSCI.3689-11.2012 22219283 10.1523/JNEUROSCI.3689-11.2012 PMC6621313

[121] Torta DM, Cauda F (2011) Different functions in the cingulate cortex, a meta-analytic connectivity modeling study. Neuroimage 56: 2157–2172 doi:10.1016/j.neuroimage.2011.03.066 21459151 10.1016/j.neuroimage.2011.03.066

[122] Sandak R, Mencl WE, Frost SJ, Rueckl JG, Katz L, et al (2004) The neurobiology of adaptive learning n reading: A contrast of different training conditions. Cogn Affect Behav Neurosci 4: 67–88. 15259890 10.3758/cabn.4.1.67

[123] Poldrack RA, Fletcher PC, Henson RN, Worsley KJ, Brett M, et al (2008) Guidelines for reporting an fMRI study. Neuroimage 40: 409–414 doi:10.1016/j.neuroimage.2007.11.048 18191585 10.1016/j.neuroimage.2007.11.048 PMC2287206

[124] Koyama MS, Kelly C, Shehzad Z, Penesetti D, Castellanos FX, et al (2010) Reading networks at rest. Cereb cortex 20: 2549–2559 doi:10.1093/cercor/bhq005 20139150 10.1093/cercor/bhq005 PMC2981020

[125] Clements-Stephens AM, Materek AD, Eason SH, Scarborough HS, Pugh KR, et al (2012) Neural circuitry associated with two different approaches to novel word learning. Dev Cogn Neurosci 2 Suppl 1S99–113 doi:10.1016/j.dcn.2011.06.001 22682916 10.1016/j.dcn.2011.06.001 PMC3295245

[126] Richards TL (2001) Functional magnetic resonance imaging and spectroscopic imaging of the brain: Application of fMRI and fMRS to reading disabilities and education. Learn Disabil Q 24: 189–203.

[127] Blachman BA, Schatschneider C, Fletcher JM, Clonan SM (2003) Early reading intervention: A classroom prevention study and a remediation study. In: Foorman BR, editor. Preventing and Remediating Reading Difficulties: Bringing Science to Scale. Timonium: York Press. pp. 253–271.

[128] Mathes PG, Denton C, Fletcher JM, Anthony JL, Francis DJ, et al (2005) The effects of theoretically different instruction and student characteristics on the skills of struggling readers. Read Res Q 40: 148–182 doi:10.1598/RRQ.40.2.2

[129] Berninger VW, Winn WD, Stock P, Abbott RD, Eschen K, et al (2007) Tier 3 specialized writing instruction for students with dyslexia. Read Writ 21: 95–129 doi:10.1007/s11145-007-9066-x

[130] Winn W, Berninger V, Richards T, Aylward E, Stock P, et al (2006) Effects of nonverbal problem solving treatment on skills for externalizing visual representation in upper elementary grade students with and without dyslexia. J Educ Comput Res 34: 381–404.

[131] McGuiness C, McGuiness D, McGuiness G (1996) Phono-Graphix: A new method for remediating reading difficulties. Ann Dyslexia 46: 73–96. 24234268 10.1007/BF02648172

[132] Ihnot C, Mastoff J, Gavin J, Hendrickson L (2001) Read naturally. St. Paul, MN: Read Naturally.

[133] Avrit K, Allen C, Carlsen K, Gross M, Pierce D, et al.. (2006) Take flight: A comprehensive intervention for students with dyslexia. Dallas: Texas Scottish Rite Hospital for Children.

[134] Vaughn S, Cirino PT, Wanzek J, Wexler J, Fletcher JM, et al (2010) Response to intervention for middle school students with reading difficulties: Effects of a primary and secondary intervention. School Psych Rev 39: 3–21. 21479079 PMC3072689

[135] Kame'enui E, Simmons D (2003) Early reading intervention. Scott-Foresman Publishing.

[136] May P, Vieluf U, Malitzky V (2000) Hamburger Schreibprobe: Diagnose orthographischer Kompetenz. Hamburg, Germany: Verlag für pädagogische Medien.

[137] Lyytinen H, Ronimus M, Alanko A, Poikkeus A-M, Taanila M (2007) Early identification of dyslexia and the use of computer game-based practice to support reading acquisition. Nord Psychol 59: 109–126 doi:10.1027/1901-2276.59.2.109

[138] Lyytinen H, Erskine J, Kujala J, Ojanen E, Richardson U (2009) In search of a science-based application: a learning tool for reading acquisition. Scand J Psychol 50: 668–675 doi:10.1111/j.1467-9450.2009.00791.x 19930268 10.1111/j.1467-9450.2009.00791.x

[139] Saine NL, Lerkkanen M-K, Ahonen T, Tolvanen A, Lyytinen H (2011) Computer-assisted remedial reading intervention for school beginners at risk for reading disability. Child Dev 82: 1013–1028 doi:10.1111/j.1467-8624.2011.01580.x 21418055 10.1111/j.1467-8624.2011.01580.x

